# Efficacy, safety, and retention rate of extended-release divalproex versus conventional delayed-release divalproex: A protocol for systematic review and meta-analysis

**DOI:** 10.1097/MD.0000000000028290

**Published:** 2021-12-17

**Authors:** Chen Qi Zhang, Xue Yang Bai, Yong Wan, Hong Yan Li, Hongbin Sun

**Affiliations:** a Department of Special Medical, Chengdu BOE Hospital, Chengdu, Sichuan Province, China; b Department of Neurology, The Air Force Hospital of Western Theater Command, Chengdu, Sichuan Province, China.

**Keywords:** divalproex, efficacy, extended release, meta-analysis, safety

## Abstract

**Background:**

A novel once-daily divalproex-extended release (VPA-ER) dose formulation has been developed, which prolongs therapeutic serum levels compared with that of twice-daily conventional divalproex-delayed release (VPA-DR). Currently, there is lack of meta-analysis focusing on drug retention rate between VPA-ER and VPA-DR. Thus, our study is the first one that aims to systematically examine and compare the efficacy, safety, and retention rate of VPA-ER and VPA-DR.

**Methods:**

Online databases including MEDLINE via PubMed, Cochrane Library, Embase (up to October 30, 2021) will be applied for literature screen. We will conduct meta-analysis by using Stata16.0 software.

**Results:**

This study aims to evaluate the efficacy, safety, and drug retention rate of VPA-ER versus conventional VPA-DR

**Conclusion:**

Once-daily VPA-ER may present a positive efficacy and not increase the incidence of AEs and has a higher retention rate for patients, which can be used as a substitute for conventional VPA-DR.

INPLASY registration number: INPLASY2021110090

(DOI: 10.37766/inplasy2021.11.0090).

## Abstract 2

Supplemental Digital Content is available in the text

## 1 Introduction

Valproic acid and its derivatives, including valproate sodium (sodium valproate), divalproex and divalproex sodium are all known as valproate (VPA). VPA is the most commonly applied first-generation broad-spectrum antiepileptic drug (AED) to generalized and focal epilepsies in children and adults approved by the Food and Drug Administration (FDA). Divalproex has been approved for use in the US since 1983.^[1]^ Additionally, it has a broad spectrum on the treatment of bipolar depression and rapid cycling, psychotic symptoms, impulsive aggression, post-traumatic stress disorder (PTSD), neuropathic pain, and the prophylaxis of migraine headaches.^[2–7]^

VPA has been approved in several formulations, including the original delayed-release tablet (e.g., Depakote), enteric-coated particles, sprinkle capsules, sustained-release (e.g., Depakine Chrono) and a more recently approved, extended-release tablet (e.g., Depakote ER). In our study, divalproex-extended release (VPA-ER) and divalproex-delayed release (VPA-DR) will be defined as all extended-release and delayed-release formulations of VPA, respectively. In ER formulations, the dosing interval is usually extended to minimize the dosing frequency.^[8]^ In addition, they can potentially minimize the spikes in the maximum plasma concentrations (Cmax) at a steady-state and maintain the relatively constant or flat plasma drug concentration. Moreover, they can minimize concentration-related AEs.^[9]^ Compared with the standard twice-daily (BID) DR formulation, once-daily VPA-ER significantly stabilizes serum levels without marked peak-to-trough fluctuations, reduces dosing frequency and the possibility of dosing flexibility, which improves patient compliance, satisfaction and ultimately the quality of life.^[7,10]^

Although once-daily formulation is more convenient than multiple doses per day, potential sub-therapeutic concentrations following delayed or missed doses should be concerned. Once-daily formulation is unable to improve therapeutic coverage cause because it cannot maintain the effective drug concentration in biological fluids and tissues pharmacokinetically. The risk of breakthrough seizure is higher in once-daily AED administration than that of twice-daily administration.^[11,12]^

Currently, ER formulations are preferred to the treatment of EP and prevention of MA due to the better compliance, convenience, and flat plasma concentration with time. To our knowledge, comparison and conversion studies about VPA-ER and conventional VPA-DR are lacked. Small-sample studies analyzing the safety and efficacy of VPA-ER in different populations remain controversial and do not focus on medicine retention rate. Therefore, our study aims to systematically review and analyze the safety, efficacy and retention rate of VPA-ER and VPA-DR by meta-analysis.

## 2 Methods

### 2.1 Study registration

This protocol of this study was registered with the International Platform of Registered Systematic Review and Meta-analysis Protocols (INPLASY), the registration number is INPLASY2021110090 and the DOI number is 10.37766/inplasy2021.11.0090 (https://inplasy.com/inplasy-2021–11–0090/).

### 2.2 Selection criteria

#### 2.2.1 Study population

We will include any participants receiving VPA-ER and VPA-DR monotherapy without limitations on disease, age, gender, race, treatment time and region of the enrolled.

#### 2.2.2 Interventions and comparators

The treatment groups were defined as patients who were treated with extended-release formulations of VPA monotherapy, and the control group will be assigned with oral delayed-release formulations of VPA or placebo.

#### 2.2.3 Outcomes assessment

Clinical efficacy assessed in patients with different diseases (e.g., in epilepsy patients, at least 50% reduction in seizure frequency or complete seizure freedom m was achieved) will be the primary outcome indicator. The secondary outcome indicators will be safety assessment (e.g., the incidence of adverse events) and drug retention rate assessment.

#### 2.2.4 Study design

We aim to likely include randomized controlled trails single-blind/double-blind, crossover, open labeled trials reporting the efficacy, safety, medicine compliance of VPA-ER and the control were included. Unclearly defined divalproex-ER and literatures with missing or repeated data will be excluded. Even though we don’t have restrictions on language and region, but the search object was restricted to human. The protocol of the meta-analysis will be developed according to the Preferred Reporting Items for Systematic Reviews and Meta-Analysis Protocols (PRISMA-P) 2015 statement guidelines.^[13]^

### 2.3 Search strategy

We will search articles in three electronic database including PubMed, EMBASE and Cochrane Library without language limitation. All the publications until October 30, 2021 will be searched without any restriction of countries or article type through a combination of MeSH words and term words, including “Valproic acid” [Mesh], “divalpro∗,” “Valpro∗,” “Depak?e,” “Semisodium Valproate,” and “Modified-release,” “Delayed-release,” “Extended-release,” “sustained-release,” “prolonged-release” “controlled-release,” and “treatment” [Mesh], “Therapeutics[Mesh],” “therap∗,” “Treatment∗.” Moreover, reference list of all selected articles will independently be screened to identify additional studies left out in the initial search.

### 2.4 Study selection and data extraction

We will perform Endnote X9 literature management software for all procession of the screening records. Two research (ZCQ and BXY) were independently responsible for searching eligible RCTs in online Databases and data extraction, and any disagreement was solved by discussion with the third senior reviewer (LHY or SHB). The following data will be extracted: author, year of publication, total number of people included in the study, mean age, gender, original diagnostic criteria, study period, doses of progesterone and time of application, treatment and control intervention, main outcome measures and AEs.

### 2.5 Risk of bias assessment

The quality of included literatures was independently reviewed by two reviewers respectively using the Cochrane handbooks (http://community.cochrane.org/handbook). Including the following 7 aspects: random sequence generation, allocation concealment, blinding of participants and personnel, blinding of assessors, incomplete outcome data and selective outcomes reporting.

### 2.6 Statistical analysis

Stata 16.0 software (Stata Corporation, College Station, TX) will be applied for statistical analyses. A dichotomous analysis with the risk ratios (RRs) and 95% confidence intervals (CIs) were calculated to compare differences between groups. While standardized mean difference (SMD) with the 95% CI as an effect size was measured for continuous data. The heterogeneity was assessed by the Cochran's Q test and *I*^2^ tests. If *P* < .05 and *I*^2^ > 50%, data were analyzed using a random effect model. Otherwise, they were analyzed using a fixed effect model. Two tailed *P* < .05 was considered statistically significant. If necessary, a leave-one-out sensitivity analysis will be performed to evaluate the main trials demonstrating a substantial impact on the inter-study heterogeneity. Besides, we plan to conduct subgroup analyses to evaluate the influence of different disease populations on clinical efficacy and safety and the cause of withdrawal on retention rate.

### 2.7 Confidence in cumulative evidence

The Grading of Recommendations Assessment will be performed. To evaluate the results of the analysis, the domains include, the domains include the following 5 aspects (risks of bias, indirection, inconsistency, imprecision, and publication bias) are divided into 4 levels: Very low, low, medium, and high.

### 2.8 Ethics and dissemination

This study will not require an ethical approval since no subjects were recruited.

## 3 Discussion

VPA is a weak sodium channel blocker that produce weak inhibitors of enzymes to inactivate gamma-aminobutyric acid (GABA) like aminobutyric aminotransferase.^[14]^ In addition, VPA can regulate serotonergic and glutamatergic transmission, energy metabolism, neuronal membrane lipid synthesis, neurotrophic and neuroplastic effects, etc.^[15,16]^ Many AEDs, including VPA, have short half-lives that must be administered several times daily with large fluctuations in peak-to-trough plasma concentrations,^[11]^ resulting in poor pharmacokinetic (PK) properties, AEs and unsatisfactory adherence.^[8]^

Once-daily VPA-ER is featured by the hydrophilic polymer matrix controlled-release tablet system, which allows for the slow release of drugs in the stomach, small intestine, and large intestine for 18 to 24 hours,^[17]^ which is differenced with the standard twice-daily (BID) DR formulation. Reducing dosing frequency per day can result in a significant increase in treatment compliance.^[1]^ A previous study had showed a similar or better efficacy of divalprex-ER on seizure and mood disorders in 9 open labeled trials.^[18]^ As well as an observational study involving 359 epilepsy patients demonstrated that over 95% of patients administrated with once-daily evening dosing of valproate sustained release minitablets have a good compliance/acceptance.^[19]^

However, the positive effect of VPA-ER had only been reported in several clinical studies which including open labeled trials and observational research. At present, there is lack of meta-analysis focusing on retention rate of patients treated with VPA-ER versus VPA-DR and it requires further investigation and standardized ways to evaluate the effects of VPA-ER in different disease patients. Thus, we aim to likely analyze the efficacy, safety and drug retention rate in patients treated with VPA-ER based on RCTs and offer direct evidence to aid physicians in making clinical decisions (Supplement 1 Search Strategy, http://links.lww.com/MD2/A757).

## Author contributions

**Conceptualization:** Chenqi Zhang, Hongbin Sun.

**Data curation:** Chenqi Zhang, Xueyang Bai.

**Formal analysis:** Chenqi Zhang, Xueyang Bai.

**Funding acquisition:** Chenqi Zhang.

**Investigation:** Yong Wan.

**Methodology:** Hongbin Sun.

**Software:** Yong Wan.

**Supervision:** Hongyan Li, Hongbin Sun.

**Validation:** Hongyan Li, Hongbin Sun.

**Writing – original draft:** Chenqi Zhang.
